# Unlicensed/Off-Label Drug Prescriptions at Hospital Discharge in Children: An Observational Study Using Routinely Collected Health Data

**DOI:** 10.3390/healthcare12020208

**Published:** 2024-01-15

**Authors:** Elham Jaberi, Inesse Boussaha, Xavier Dode, Guillaume Grenet, Behrouz Kassai, Kim An Nguyen

**Affiliations:** 1 UMR CNRS 5558, Laboratoire de Biométrie et Biologie Humaine, Équipe Évaluation et Modélisation des Effets Thérapeutiques, rue Guillaume-Paradin, BP8071, CEDEX 08, F-69376 Lyon, France; 2 Department of Clinical Epidemiology, Hospices Civils de Lyon, CIC 1407 de Lyon, Inserm, CHU-Lyon, F-69677 Bron, France; 3 Pharmacy Department, Hospices Civils de Lyon University Hospital, F-69002 Lyon, France; 4 Department of Pharmacotoxicology, Hospices Civils de Lyon, CHU-Lyon, F-69677 Bron, France

**Keywords:** children, drug prescription, discharge, unlicensed, off-label

## Abstract

Background: Unlicensed and off-label (UL/OL) prescriptions have been associated with an increased risk of drug-related problems. Data of their prevalence at hospital discharge remain insufficient. We aimed to describe the prevalence of UL/OL drugs in outpatient prescriptions at discharge in children. Methods: We conducted a retrospective study using the routinely collected health data of children at discharge from 2014 to 2016. The primary reference source for determining licensed labelling was the summaries of product characteristics (SPCs) in a French industry-independent formulary named Thériaque. We described the characteristics of UL/OL prescriptions at discharge and looked for predictors of UL/OL prescriptions. Results: We included 2536 prescriptions of 479 children. Licensed, OL, and UL prescriptions accounted for 58.6% (95% CI: 56.7–60.5), 39.2% (95% CI: 37.3–41.1), and 2.3% (95% CI: 1.7–2.9), respectively. A total of 323 (74%) children received at least one UL/OL drug. Among the licensed drugs, bronchodilators (8.8%) and analgesics (8.6%), and among the OL drugs, antibiotics (2.8%), were the most prescribed. The younger age of the children and higher number of drugs they received increased the probability of UL/OL prescriptions (unadjusted *p*-value of ≤0.05). Conclusion: The prevalence of UL/OL prescriptions is about 40% at discharge from a pediatric university hospital in France.

## 1. Introduction

Marketing authorization (MA) based on randomized controlled trials (RCTs) is mandatory for ensuring the safety, the quality, and the efficacy of a drug [1]. While most of the MA application files of a medicinal product comprise clinical trials, about 50% of the drugs used in children have been studied only in adults and sometimes for a different indication [2,3]. Many drugs administered to children in hospitals either lack approval for use in children or are prescribed outside the boundaries of their product license, which is known as off-label (OL) prescribing [4]. This issue results from the scarcity of clinical trials involving this population, coupled with the practical challenges and ethical considerations associated with including children in clinical research [5]. The information accessible to pediatricians might not consistently be as comprehensive or robust as when prescribing a drug that is licensed for an approved indication [6,7]. However, globally, there is widespread use of OL and unlicensed (UL) prescribing for children. Clinicians argue that such prescriptions are almost unavoidable, given the absence of RCTs for many drugs in the pediatric population and the need to address serious diseases or urgent situations when no licensed alternatives are available [3].

A systematic review of studies on UL/OL drug use in hospitalized children showed that 12.2% to 70.6% concerned OL drugs and 0.2% to 47.9% concerned UL drugs. Children who received at least one OL and/or UL drug ranged from 42.0% to 100.0%, with newborns receiving most of such drugs [8]. This has led to concerns that children can receive drugs at dosages that either lack efficacy or exhibit safety problems. Comparing to licensed prescriptions, some studies showed that UL/OL prescriptions increased about two-fold the risk of adverse drug reactions (ADRs) in hospitalized children [4].

Appropriate medication at hospital discharge is a vital aspect of an enhanced transition from inpatient to outpatient care in children experiencing different illnesses [9]. However, the hospital discharge process exhibits considerable variability and a substandard quality of care, frequently characterized by errors, omissions, duplications, and inconsistencies in pediatric discharge care [10,11,12]. Such discharge care negatively impacts patients’ health and increases the probability of subsequent hospital readmissions. Hospital readmission rates serve as a measure of the quality of care provided in children’s hospitals. One major cause of hospital readmission is dysfunctions in coordinated pathways between community and hospital care that can cause adverse drug events [13,14]. According to a study, the proportion of prescriptions at discharge with at least one drug-related problem (DRP) was 65.7%. DRPs included inappropriate drug selection (35.6%), wrong time of dosing relative to meals (35.6%), inappropriate dosage form (9.3%), inappropriate indication (7.1%), and drug–drug interactions (0.3%) [15]. Dick et al. showed that out of 308 pediatric patients receiving 777 prescriptions at hospital discharge, 49% concerned an UL or OL use, of which 76% were OL [16]. While the majority of studies assessing the evaluation of UL/OL drug prescriptions were based on inpatient [17] or outpatient populations [18], data concerning medication at discharge, particularly in children, are insufficient. For our best search, we did not find any study concerning UL/OL prescriptions in children at hospital discharge in France [19]. We aimed to determine the rate of UL/OL prescriptions and their predictive factors in hospitalized children at discharge.

## 2. Materials and Methods

### 2.1. Study Design and Setting

This was an observational retrospective study using routinely collected numeric hospital records of discharge prescriptions data, which were only available from a sample of the Lyon EREMI cohort. EREMI is a multi-center prospective study which evaluates the relationship between UL/OL drug use and ADRs in hospitalized children in France [20].

A prescription was defined as a drug with a dosage, frequency, route of administration, and duration for one patient. Therefore, a patient could have more than one prescription of the same drug.

All further explorations based only on the anonymous EREMI database were approved by the ethics committee (authorization obtained on 20 July 2016 (CECIC Rhône-Alpes-Auvergne, Clermont-Ferrand, IRB5891)) according to the General Data Protection Regulation (GDPR) in Europe. The extraction and analysis of the data took place in 2022.

### 2.2. Data Collection

We managed and analyzed all discharge prescription data available from January 2013 to December 2016 in numerical format of the children recruited for the EREMI study. Considering that some of the patients were hospitalized more than once, if a patient had more than one discharge prescription, data from all hospital discharge medication during the study period were included in the analysis. Demographic patient data included age, sex, weight, and height. We extracted medication data with generic names, dosage, route of administration, frequency, and duration of treatment. We limited the scope of our study to drugs and vitamins. In France, some vitamins like vitamin D are considered medicinal and require prescriptions. We included only vitamins in medical prescriptions, excluding those available over the counter. Other health products, such as blood-derived products, nutrition, contrast products, oxygen or other gases, nutrient infusions or non-drug infusions, and non-medical topical products such as moisturizers, ointments, and lotions, were excluded.

### 2.3. Determining UL/OL Drugs

UL/OL prescribing was identified and categorized according to the definition of Turner et al. [4]. The categories of UL use included the following: the modified use of licensed drugs (for example, crushing tablets to create a suspension), drugs that are licensed but formulated under a specific license (such as a liquid version of a tablet-only licensed drug), the use of chemical substances as drugs in the absence of pharmaceutical-grade preparations, drugs administered before obtaining a license, imported drugs (those brought in from a country where they hold a license), and drugs lacking any product license. Administration of a drug outside a defined age range, use of a different route, use of a different dosage, dosing frequency or duration of use, drugs unavailable in pediatric formulations, and prescriptions for a different indication were categories of OL use.

Two trained researchers manually and independently verified the license status of every prescription based on the summaries of product characteristics (SPCs) in a French independent formulary named Thériaque [21]. Disagreements were solved by consensus.

### 2.4. Sample Size

The sample size for this study was bound by the design of the EREMI cohort. The sample size for EREMI was previously described [20]. Briefly, it was based on a test of comparison of two proportions between two groups. In all, 1500 prescriptions by group (a total of 3000) were deemed necessary to show an Odds Ratio (OR) = 1.65 with 95% confidence that the estimate of this OR will be between 1.2 and 2.2, for the underlying hypothesis of a risk of ADRs of 5% in the group of “licensed drugs” and 8% in the group of “UL/OL drugs” (two-sided alpha = 0.05; power = 90%). A total of 4032 children were included in EREMI with more than 10 thousand prescriptions.

### 2.5. Population of the Study

The EREMI study included 4032 participants from pediatric wards in Lyon between 2013 and 2016. The wards were nephrology, gastro-enterology, endocrinology, metabolic and hereditary diseases, child psychiatry, neurology, pneumology, pediatric cardiology, neurology. Among those, we retrieved 939 children with prescription at discharge. We were able to include 479 patients from 2014 to 2016. For feasibility and due to the available human resources at this time, we did not include 460 patients from 2013. The neonatal department was not included in this study.

### 2.6. Data Processing and Statistical Analysis

#### 2.6.1. Epidemiological Characteristics of UL/OL Prescriptions

We determined the number of individuals who were prescribed and received at least one UL/OL drug. The prevalence of UL/OL drug use was calculated from the number of children who were prescribed at least one UL/OL drug at hospital discharge, divided by the total number of included children. We described the 12 most commonly prescribed drugs and their license status. We identified the 5 most commonly prescribed UL/OL drugs.

#### 2.6.2. Subgroup Analyses

The prevalence of UL/OL drugs was determined in different age subgroups. We used the International Conference on Harmonization of Technical Requirements for Registration of Pharmaceutical for Human Use (ICH) Harmonized Tripartite Guideline E11 for a clinical investigation of medicinal products in the pediatric population [22] to define four age groups, including term newborn infants (0–27 days); infant toddlers (1–23 months); children (2–11 years); and adolescents (12–18 years).

#### 2.6.3. Search for Predictors of UL/OL Prescriptions

To identify determinants of UL/OL prescriptions, we conducted multivariate regression. Independent variables of interest included the age of the children and the number of drugs they received. A threshold of *p*-value ≤ 0.05 was set to indicate statistical significance of the variable effect, without adjustment of the number of analyses.

Descriptive analyses were performed using Microsoft Office Excel (2016) [23]. We used R software [24] version 4.2.1 to carry out the multivariate regression.

## 3. Results

### 3.1. The Characteristics of the Included Children

**Figure 1:**
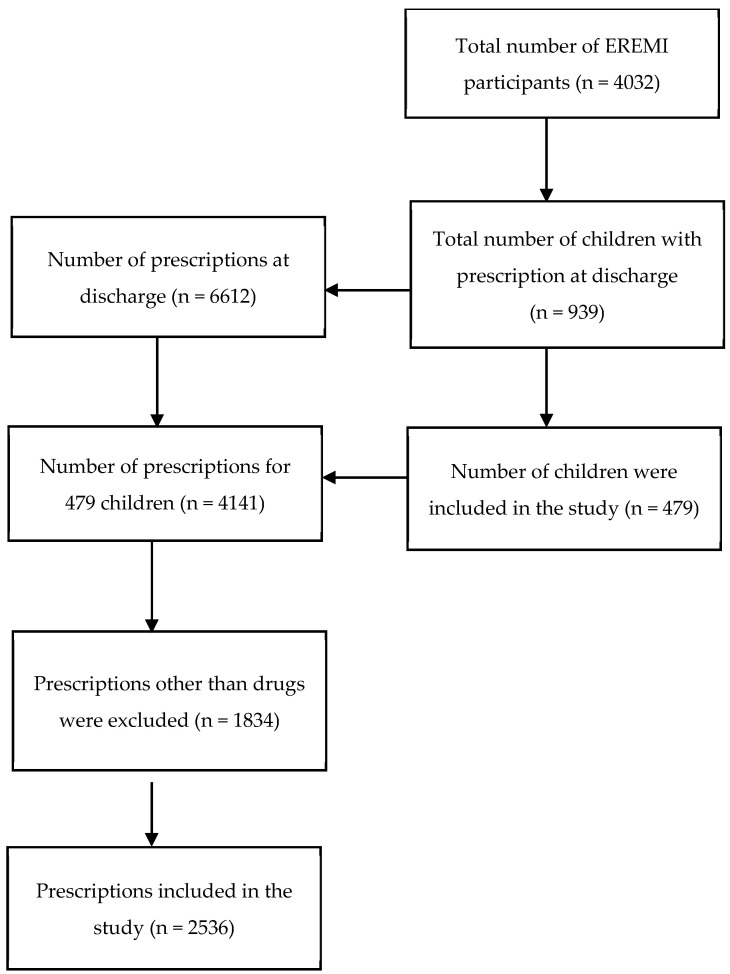
Flow chart of inclusion.

Of the 4032 participants in EREMI, 939 had a prescription at discharge. Of the 479 children that were in the study, data were available for 4141 prescriptions. Excluding 1834 non-drug prescriptions, 2536 drug prescriptions were included for analysis (Figure 1).

The gender ratio was 1.4 (265 males per 194 females). The median age was 5 years (range 3–10). The median weight of the patients at discharge was 19.9 kg (range 12.3–32.1). The median prescription number per child was 3 (range 1–6) (Table 1). Because the number of children who took fewer prescriptions was higher, the median was lower than the mean (additional information can be seen in Figure 1).

### 3.2. Primary Outcome

UL/OL drug use for children at discharge was 41.5% (95% CI: 39.5–43.3). Licensed prescriptions were 58.6% (95% CI: 56.7–60.5); OL accounted for 39.2% (95% CI: 37.3–41.1) and UL was 2.3% (95% CI:1.7–2.9). A total of 323 (74%) children received at least one UL/OL drug (Table 2).

Among the 2536 prescriptions, no drug exceeded 10% of the total prescriptions, and all of the most commonly prescribed drugs were licensed. Bronchodilators (salbutamol) and analgesics (paracetamol) were the most prescribed classes according to their license. They were followed by vitamins (A, D, ADEC) and corticosteroids (prednisolone and mometasone) (Table 3), of which about 10% (95% CI: 5.8–15.6) and 13.5% (95% CI: 8.8–15.4) of their prescriptions were used OL, respectively.

All OL prescriptions were identified as OL for age. The most commonly prescribed OL drugs were antibiotics (amoxicillin and clavulanic acid) and laxatives (macrogol) (Table 4).

All of the UL drugs were modified formulations of licensed drugs by local pharmacies or for temporary and specific authorization use. Among them, vitamin A and melatonin were the most common (Table 5).

In the multivariate analysis, the age of the children (OR = 1.88, 95% CI: 1.03–3.46, *p*-value = 0.04) and the number of drugs they received (OR = 11.01, 95% CI: 6.27–19.35, *p*-value < 0001) had significant effects on the probability of UL/OL prescriptions. The younger age of the children and higher number of drugs they received increased the probability of UL/OL prescriptions.

## 4. Discussion

To the best of our knowledge, this is the first time that the use of UL/OL drugs in children at hospital discharge has been reported from a French pediatric university hospital. The findings of this study show that about 41.5% (95% CI: 39.5–43.3) of the prescribed drugs for children at discharge were UL/OL. From this number, 57 (2.3%) of the prescribed drugs were UL, which were local pharmacy modifications of licensed drugs or based on temporary and specific authorization use and 993 (39.2%) were OL with regard to the child’s age. At least one UL/OL drug was prescribed for 74% of the children. The highest absolute number of OL prescriptions was found for antibiotics (amoxicillin and clavulanic acid).

Similar to our results, previous studies have shown that UL/OL drug use in children at discharge is common. However, the UL/OL classification methods with respect to the primary reference source and UL/OL definition varied, making the results difficult to compare. In general, UL/OL prescription rates for children at discharge ranged from 12% to 50% [16,25,26,27].

In our study, all OL prescriptions were OL for age, which was a finding similar to a previous study [28]. On the other hand, other studies reported that the indication [16] and the route of administration in a premature group [29] were the most common reasons for OL prescriptions. Our result was underestimated because of the lack of indication evaluation.

Our results suffered from an unbalanced distribution of patients across different age groups. The administration of UL/OL drugs occurred more frequently among children aged 2–11 years compared to other age groups. Within this age category, the number of prescriptions per patient was also the highest. This trend is likely attributed to the higher morbidity rate among children in this age range. As children get older, the risk of multiple therapies due to chronic illnesses increases. Our results were in accordance with one report from another French hospital, which showed that children (2–11 years old) and adolescents (12–18 years old) received the highest number of UL/OL prescriptions during hospitalization (45% and 46.8%, respectively) [30].

All UL prescriptions (2.3%) in our study were due to modified formulations or the temporary authorization of drug use for specific indications in pediatrics during the study period. Previous studies conducted in France reported UL use to be rare: 3.2%, 5.2%, and 4% of all prescriptions in pediatric hospitals, neonatal intensive care units (NICUs), and office-based pediatricians, respectively [30,31,32].

For the risk factors of UL/OL drug use, similar to some studies [28,33,34], we found that younger age of the children and higher number of drugs they received increased the probability of UL/OL prescriptions. However, due to the small sample size in a single center, these results must be interpreted with caution.

Our single-center study and small number of patients make the generalizability of our results difficult. We only studied first OL prescriptions for age and then for the dosage and route of administration of all licensed drugs. We could not combine two or more characteristics of OL status. We could not evaluate the indication and interaction in this study. The non-inclusion of these criteria increased the risk of underestimating the prevalence of OL prescriptions. Since all of the EREMI prescriptions at discharge, particularly the prescription data at discharge in the year 2013, were not included in the study, there was a risk of potential selection bias to time of entry. It could be that earlier subjects were different to later subjects. Finally, it is also worth noting that because it was not possible to verify the indication of UL/OL use by the children, the lowest approved age was considered when the drug had more than one indication as well as different minimum ages for each indication.

Despite these limitations, our study has some strengths. First, there is little information in the literature regarding the discharge data, which we highlighted herein, considering the recurrence of UL/OL drug prescriptions for children in all healthcare levels. As previously mentioned, we are unaware of any studies on hospital-discharged children in France, with this being the first of this kind. Additionally, with regard to the importance of transitioning from hospital to home [35] in particular, this study’s results may be useful for both clinicians and policymakers in planning appropriate discharge transitions.

### Implications for Practice and Research

To better survey the UL/OL use of drugs in children in real time, we believe that future research should focus on developing an algorithm tool for automatic UL/OL classification because manual classification, as we used in this study, was labored and very time-consuming for us.

Clinical trials offer reliable evidence of the impact of treatments through rigorous controlled testing of interventions on human subjects [5]. Significant changes in drug labeling for pediatric patients demonstrate the need for specific pediatric dosing, taking into account the growth and maturation stages of these patients [4]. The responsibility for demonstrating the safety and efficacy of medicinal products, as well as determining their appropriate dosage, lies with drug regulatory authorities [36]. Despite advancements in current regulations and initiatives aimed at enhancing the scope, quantity, and quality of trials involving the pediatric population, challenges such as recruitment and ethical obstacles persist in initiating and conducting clinical trials in children. Addressing these challenges is essential to significantly speed up equitable access to evidence-based treatments for children. Professionals dedicated to providing optimal medical care for children recognize the necessity of increasing the future availability of rigorously validated medicinal treatments. Due the enforcement of obtaining pediatric information by regulatory agencies, many efforts are still needed to provide children with access to drugs with the same scientific support as drugs for adults.

Creating formulations specific for children necessitates collaboration between the public and private sectors. Enhanced support and cooperation among key stakeholders, including regulatory authorities, pharmaceutical industries, the scientific community, clinicians, and the public at both national and international levels, are vital for attaining this success [5]. A more efficient operational structure for conducting clinical trials should align with the continually advancing regulatory framework, which encourages investment in pediatric clinical trials. Utilizing technological methods, improving electronic medical record systems, and implementing community approaches that actively involve input from physicians, researchers, and patients could provide a lasting solution for recruiting participants in pediatric studies. Both the United States (US) and the European Union (EU) have observed a rise in the number of clinical trials involving children. In the US, numerous studies, encouraged by the Best Pharmaceuticals for Children Act (BPCA) and the Pediatric Research Equity Act (PREA), have been conducted with children, which resulted in adjustments in the labeling of several drugs. The European Commission has also implemented regulatory actions to promote the licensing of drugs in children [37]. Adequate resources for pediatric clinical trials are essential if children are to be given drugs that are safe and effective for use in their age group.

Additionally, while it is recognized that safety data in adults can be employed to anticipate certain adverse effects in children, there is still a lack of information on adverse reactions and potential long-term toxicity from medicines in children. Controversial data and limited evidence exist regarding the risks of serious adverse reactions from the use of UL/OL drugs in children. Further research in this field should focus on the burden of harm caused by drugs, particularly inappropriate UL/OL drug use. Otherwise, appropriate formulation for age is the main reason for using UL/OL drugs in children. Recently, in France, the Agence Nationale de Sécurité du Médicament et des Produits de Santé (ANSM) published their position endorsing appropriate drug use on an individual level, especially in frail populations such as children. They highlighted the inappropriate UL/OL use of drugs as one of the main concerns and called for collective actions [38].

The process of hospital discharge involves multiple disciplines, during which patients are provided with complex medical information and follow-up instructions [39]. However, there is a lack of high-quality evidence throughout the literature on how to improve the discharge process in children. One of the most appropriate policies to improve discharge medication requires a deeper understanding of the mechanisms through which readmissions occur. In particular, taking the initiative to explore the drug-related predictors of readmissions in order to define appropriate interventions is basically needed [40,41]. Further research on hospital discharge and the transition from hospital to home treatment is deemed necessary to evaluate inappropriate UL/OL prescriptions and their consequences in children. 

## 5. Conclusions

We showed that the prevalence of UL/OL prescriptions was about 40%, and 74% of children received at least one UL/OL drug at discharge from a pediatric university hospital in France. Younger age in the children and a greater number of drugs they received increased the probability of UL/OL drug use.

## Figures and Tables

**Table 1:** Demographics and general prescription characteristics by age class.

	<27 Days	28 Days–23 Months	2–11 Years	12–18 Years	Total
**Demographics**					
Number of children discharged (%)	2 (0.4)	138 (27.2)	298 (58.9)	68 (13.4)	479
Gender M ^1^/F ^2^	2/0	81/55	159/125	32/30	265/194
Median weight at discharge (kg) (IQR ^3^)	4.1 (4.1–4.1)	8.6 (7.2–9.7)	19.8 (14.4–27.8)	39 (32.5–46.6)	19.9 (12.3–32.1)
Mean weight at discharge (kg) (SD ^4^)	4.1 (0)	8.7 (3)	22.4 (11.7)	39.7 (13.4)	23.7 (14.5)
**Prescription characteristics**					
Number of prescriptions	4	391	1642	499	2536
Number of products used	3	93	265	147	309
Number of prescriptions per child. Median (IQR)	2	2 (1–4)	3 (1–6)	4 (2–9)	3 (1–6)

^1^ male; ^2^ female; ^3^ interquartile range; ^4^ standard deviation.

**Table 2:** Primary outcome.

	<27 Days	28 Days–23 Months	2–11 Years	12–18 Years	Total
Number of OL ^1^ prescriptions (%)	2 (50)	134 (34.3)	660 (40.2)	195 (39.1)	993 (39.2)
Number of UL ^2^ prescriptions (%)	0	11 (2.8)	36 (2.3)	10 (2)	57 (2.3)
Number of children with OL or UL prescriptions (%)	2 (100)	70 (51)	208 (70)	53 (78)	323 (74)

^1^ off-label; ^2^ unlicensed.

**Table 3:** Most commonly prescribed drugs and their labeling status (% of all prescriptions and 95% CI).

Drug Name	Total Prescriptions N ^1^, 2536	Percentage (95% CI ^2^)	Labeling Status
Salbutamol	222	8.8 (7.7–9.9)	Licensed
Paracetamol	217	8.6 (7.5–9.7)	Licensed
Prednisolone	129	5.1 (4.3–6.0)	Licensed
Amoxicillin and clavulanic acid	86	3.4 (2.7–4.2)	Licensed
Esomeprazole	64	2.5 (1.9–3.2)	Licensed
Sulfamethoxazole and trimethoprim	63	2.5 (1.9–3.2)	Licensed
Vitamin D	61	2.4 (1.8–3.1)	Licensed
Macrogol	60	2.4 (1.8–3.0)	Licensed
Pancreatin	58	2.3 (1.7–2.9)	Licensed
Vitamin A	56	2.2 (1.7–2.9)	Licensed
Mometasone	49	2.0 (1.4–2.5)	Licensed
Vitamin (ADEC)	44	1.7 (1.3–2.3)	Licensed

^1^ number of prescriptions; ^2^ confidence interval.

**Table 4:** Most commonly prescribed off-label drugs (% of all prescriptions and 95% CI).

Generic Name	Trade Name	Percentage (95% CI ^1^)	OL ^2^ Characteristics
Amoxicillin and clavulanic acid	Augmentine pdr ^3^ for oral susp. ^4^ 250 mg/5 mL—tbl. ^5^ 1 G—cap. ^6^ 500 mg	2.8 (2.2–3.5)	Weight < 40 kg
Macrogol	Forlax 10 g, pdr for oral solution in sachet	0.9 (0.6–1.4)	Age < 8 years old
Esomeprazole	Inexium tbl. 20 mg	0.7 (0.4–1.1)	Age < 18 years old
Vitamin D	Uvedose 100,000 UI oral sol. ^7^ (ampoule)	0.6 (0.4–1)	Age < 5 years old
Sulfamethoxazole and trimethoprim	Bactrim forte tbl. 800 + 160 mg	0.6 (0.4–1)	Age < 18 years old

^1^ confidence interval; ^2^ off-label; ^3^ powder; ^4^ suspension; ^5^ tablet; ^6^ capsule; ^7^ solution.

**Table 5:** Most commonly prescribed unlicensed drugs (% of all prescriptions and 95% CI).

Name	Percentage (95% CI ^1^)
Vitamin A	0.5 (0.3–0.9)
Melatonin	0.2 (0.0–0.4)
Enalapril	0.1 (0.0–0.3)
Ursodesoxycholic acid	0.08 (0.0–03)
Fludrocortisone	0.08 (0.0–0.3)

^1^ confidence interval.

## Data Availability

The data for analysis will be provided with specific requirements. The data are not available as open data.
